# Emotional Impact of Graphic Health Warnings on Tobacco Packaging: Analysis of Their Content

**DOI:** 10.5964/ejop.2885

**Published:** 2022-02-25

**Authors:** Carlos Gantiva, Miguel Sotaquirá, Vanessa Chaparro, Laura Colorado, Alejandra Gómez

**Affiliations:** 1 Department of Psychology, Universidad de los Andes, Bogotá, Colombia; 2 Department of Psychology, Universidad de San Buenaventura, Bogotá, Colombia; Glasgow Caledonian University, Glasgow, United Kingdom

**Keywords:** graphic health warnings, emotion, craving, tobacco, smoking

## Abstract

The use of graphic health warnings (GHWs) on tobacco packaging is one of the most widely used public health strategies worldwide, but there is little evidence of the emotional impact of its content and craving they generate. The objective of the present study was to evaluate the emotional and craving responses to GHW content. The study included 300 men and women of different ages (adolescents, young adults, and adults), both smokers and non-smokers. We evaluated the participants’ emotional and craving responses to 16 GHWs with different content (i.e., cancer, cardiovascular disease, abortion, and childhood illness). We analyzed the effects of sex, smoking status, and age. GHWs exhibited the capacity to elicit aversive emotional states, with low levels of arousal and high levels of dominance and did not produce craving. GHWs that showed images of cancer and abortion had the greatest emotional impact. Non-smoking adolescent females experienced the greatest emotional impact of GHWs. The results suggest that GHWs effectively reduce the attractiveness of cigarette packages and discourage consumption, and increasing the size of GHWs may favor avoidance of the package. GHWs that depicted explicit threats had a greater emotional impact, especially in women.

Tobacco kills more than 7 million people each year worldwide. Six million of these deaths are directly related to cigarette smoking, and approximately 890,000 deaths result from second-hand smoke. Tobacco consumption is currently the leading cause of lung cancer worldwide, is directly responsible for more than 29 diseases, and it is also associated with at least 50% of cardiovascular diseases (World Health Organization, 2015).

To reduce global tobacco use and demotivate new tobacco users, the World Health Organization (WHO) developed and promoted the Framework Convention on Tobacco Control (FCTC) in 2005. The FCTC was adopted by 168 nations, representing 86.5% of the countries worldwide (WHO, 2005, 2008). One of the most cost-effective methods that are defined by the FCTC for advertising about the dangers of tobacco is the use of graphic health warnings (GHWs) on tobacco packs. Currently, this strategy has been implemented by 135 countries (WHO, 2015).

Following FCTC, since 2009 Colombia introduced the use of GHWs on tobacco packages. These warnings cover 30% of the area of the main face in the pack and make use of powerful visual images and warning texts to show the negative impacts of tobacco consumption. However, their emotional impact and craving response has not yet been assessed for these warnings. In Colombia, the lifetime and last year prevalence rate of smoking was 33.3 and 12.1%, respectively; with an age onset of 17.4 years. In adolescents with ages between 12 and 17 years, the lifetime and last year prevalence rate of smoking was 6.5 and 2.4%, respectively. In young people (18–24 years), these rates increase to 31.2 and 14.6% (DANE, 2020).

Evidence suggests that GHWs are more effective among non-smokers, recent smokers (one year of consumption), and smokers with intentions to quit, compared with moderate and heavy smokers without intentions to quit (Hammond, Fong, McNeill, Borland, & Cummings, 2006; Hammond, Reid, Driezen, & Boudreau, 2012; Hammond, Thrasher, et al., 2012; Thrasher et al., 2012). GHWs that consist of images are more effective than text-only warnings (Noar et al., 2015) and GHWs compliance with the minimum size that was established by WHO (i.e., 30% of the cigarette pack) is insufficient to generate an attentional response to the health message and an emotional impact associated with avoidance of the cigarette pack (Gantiva et al., 2016; Gantiva, Palacio, Ortega, Castillo, & Ortiz, 2018). Instead, GHWs with an area covering 60% of the cigarette pack generate a greater attention and emotional response (Gantiva, Sotaquirá, et al., 2019). Recently, it has been found that GHWs equal to 85% of the cigarette pack have been shown to be more effective in increasing label awareness, motivating cessation, preventing initiation, and conveying the intended health message (Nazar et al., 2019).

The emotional impact of GHWs can be assessed using the bio-informational model that was proposed by Lang (1995). In this model, emotions are defined as predispositions to actions and are composed of three dimensions: valence, arousal, and dominance. Valence is the main dimension of emotion, which determines the activation of the appetitive motivational system (associated with pleasant emotions) and regulates approach behaviors when the goal is consummation, procreation, or nurturance. The defensive system is associated with unpleasant emotions and regulates defense mechanisms to protect the organism from threat. There is evidence that the neurophysiological substrate of valence determines its bipolar character and primacy over the other two dimensions (Bradley & Lang, 2007). Arousal refers to the energy invested in emotion (i.e., intensity of the emotional experience) and, different from valence, does not have a separate neurophysiological substrate. Lastly, the dominance dimension explains the degree of control over the perceived emotional response and implies the interruption or continuity of the behavioral response. Studies in multiple countries and cultures have corroborated the organization of emotional experience in these three dimensions (Dufey, Fernández, & Mayol, 2011; Gantiva, Barrera-Valencia, et al., 2019; Irrazábal, Aranguren, Zaldúa, & Di Giuliano, 2015; Lang, Bradley, & Cuthbert, 2008; Romo-González, González-Ochoa, Gantiva, & Campos-Uscanga, 2018; Verschuere, Crombez, & Koster, 2001; Vila et al., 2001).

Several studies have shown that tobacco-related stimuli (e.g., cigarette packages) elicit activation of the appetitive motivational system in smokers, thus favoring approach behavior (Cinciripini et al., 2006; Cui et al., 2012; Gantiva et al., 2015). Restrictions (e.g., not allowance of advertising, or the use both pictorial and text warnings illustrating the risks of smoking), that were established by the FCTC resulted in tobacco companies’ investing heavily in testing, researching, and designing more appealing cigarette packages (Wakefield, Morley, Horan, & Cummings, 2002), by constantly modifying the package design, its color combinations and textures to incentivize consumers. The effectiveness of GHWs relies on their ability to activate the defensive motivational system (i.e., aversive valence), thus allowing this strategy to compete with activation of the appetitive motivational system that is provoked by tobacco packs.

The affective image visualization paradigm is one of the most used methodologies to evaluate the emotional response to visual stimuli (Lang, Greenwald, Bradley, & Hamm, 1993). This paradigm consists of inducing emotional responses through the passive image viewing, and assesses emotional responses in the valence, arousal, and dominance dimensions with self-report or physiological measures. Using this methodology, Nascimento et al. (2008) used a set of 19 pictorial health warnings from the Brazilian tobacco control program. The GHWs elicited emotional states in the participants that ranged from neutral to very unpleasant with low to moderate arousal. The authors also found that GHWs that depicted scenes of people who smoked caused less unpleasant affective states in smokers. Furthermore, Muñoz et al. (2013) evaluated a set of 35 European tobacco-warning pictures and found that 83% of these images elicited unpleasant affective states, whereas 17% elicited pleasant states. Only 11.4% of the pictures received high arousal scores, and the authors found that women over 17 years old experienced higher levels of arousal. In both studies, GHWs that depicted explicit damage had a higher emotional impact than those that contained only symbolic imagery of danger.

However, these previous studies had three notable limitations. First, the pictures were presented using a slide projection screen; therefore, the size of the stimuli (e.g., 2.20 m × 2.50 m) was much larger than the actual size of GHWs that are presented on tobacco packs. Previous studies have shown that the size of the picture is directly related to levels of arousal, in which smaller pictures are associated with lower arousal (Codispoti & De Cesarei, 2007). Second, these studies did not focus on assessing the emotional impact of specific GHW content (e.g., cancer and cardiovascular disease). Thus, there is no evidence of the most appropriate content that is able to elicit an aversive emotional state that competes with the positive emotional state that is elicited by the cigarette package. Third, the studies did not evaluate craving (i.e., the desire to smoke) that was caused by GHWs. Therefore, the hypothesis that these stimuli do not induce craving themselves has not been tested.

Therefore, the aim of the present study was to establish the differences in the emotional and craving responses to different visual contents of the GHWs (using their real size), and to analyze the effects of sex, age, and smoking status on these responses. Based on previous studies on the emotional response to affective pictures (Lang et al., 2008), our hypothesis is that images that show more explicit harm directed towards the person will be the most effective in inducing an aversive affective state, especially in women. However, due to the small size of the warning images, a low arousal is expected (Codispoti & De Cesarei, 2007). Regarding the craving response, it is expected that GHWs do not generate craving despite the possible association with the cigarettes pack, because the content of the images inform about the negative consequences of cigarette smoking (WHO, 2015).

## Method

The ethics committee of Universidad de San Buenaventura, Bogotá, approved the experimental protocol described below.

### Participants

A convenience sample of 300 subjects (137 females and 163 males), ranging from 12 to 40 years of age (*M* = 24.27 years, *SD* = 8.66 years) and residing in Bogotá, Colombia, participated in the study. We decided to include teenagers as part of this sample, since most smokers initiate smoking when they are adolescents (Sussman, 2002). Additionally, in Colombia, the prevalence of tobacco consumption is studied from 12 years, with an onset age of 17.4 years (DANE, 2020). The sample was recruited through flyers that were posted both in different areas of the city (i.e., universities, schools, companies, factories, and libraries) and also on the Internet and social networks. For the analysis, the sample was divided into three age groups (Ministerio de Salud y Protección Social, 2018): adolescents (12–18 years old), young adults (19–26 years old), and adults (27–40 years old). Table 1 summarizes the basic demographic characteristics and smoking status of the sample.

**Table 1:** Demographic Characteristics and Smoking Status of the Participants

Characteristic	Adolescents (*n* = 100)	Young Adults (*n* = 91)	Adults (*n* = 109)	*F* or χ^2^	*p*
Age (years), *M* (*SD*)	15.58 (1.70)	21.89 (2.10)	34.22 (5.16)	785.78	< .001
Female, %	41	48.35	47.70	1.32	.51
Male, %	59	51.65	52.30		
Non-smokers, %	86	73.62	65.13	12.07	.002
Smokers, %	14	26.38	34.87		

*Note. SD* = standard deviation.

None of the participants reported current physical or psychological problems, and none of the participants were currently under pharmacological treatment for mental disorders. All of the participants had normal or corrected-to-normal vision and audition, and provided written informed consent. For participants who were younger than 18 years of age, consent was also obtained from their parents. All of the participants’ information was coded to guarantee anonymity of the data (sociodemographic characteristics, smoking status and emotional ratings).

### Stimuli

Previous studies have shown that GHWs that depicted explicit consequences had a higher emotional impact (Muñoz et al., 2013; Nascimento et al., 2008). Therefore, the GHWs that were used in the present study had to explicitly show a consequence of smoking and not simply symbolical consequences (e.g., erectile dysfunction through symbolic representation). Based on these criteria, 16 GHWs without text were selected from the set of 39 GHWs that have been used in Colombia since 2009 (see S1 in the Supplementary Materials). These GHWs were further grouped into four categories: 1) cancer, 2) cardiovascular disease, 3) abortion, and 4) childhood illness. Additionally, we selected 48 pictures (16 pleasant, 16 neutral, and 16 unpleasant) from the International Affective Picture System^1^ (IAPS; Lang et al., 2008) according to Colombian normative ratings (Gantiva, Guerra, & Vila, 2011). IAPS pictures were used as comparison stimuli and to reduce the effect of habituation to the GHWs. All of the pictures were presented in color. To simulate the size of GHWs on actual cigarette packages, the size of all of the images (i.e., both IAPS pictures and GHW pictures) was 200 × 148 pixels. Unlike with the IAPS pictures, the GHW images represent the consequences of cigarette smoking.

### Self-Report Measures

The Self-Assessment Manikin (SAM; Bradley & Lang, 1994) was used to evaluate emotional responses (i.e., valence, arousal, and dominance dimensions) to the GHW and IAPS pictures. The SAM is a pictorial nonverbal measure of emotion that consists of three affective 9-point scales. For the valence scale, the SAM ranges from a smiling, happy figure to a frowning, unhappy figure. Scores range from 1 (extremely unpleasant) to 9 (extremely pleasant), and 5 is neutral. For the arousal scale, the SAM ranges from a relaxed, sleepy figure with eyes closed to an excited, wide-eyed figure. Scores range from 1 (low arousal) to 9 (high arousal). For the dominance scale, the SAM ranges from a very small figure that represents a feeling of being controlled to a very large figure that represents being in control. Scores range from 1 (low dominance) to 9 (high dominance).

The Pictographic Assessment of Desire (PAD; Muñoz et al., 2010) was used to assess the strength of craving that was produced by the images. Similar to the SAM, it consists of a scale with five humanoid figures that represent different degrees of craving. The figure on the extreme left depicts a salivating face, representing maximum desire to consume the substance (score of 9). The figure on the extreme right depicts a relaxed face, representing no desire to consume the substance (score of 1).

### Procedure

Presentation of the pictures and SAM and PAD scales was programmed using E-Prime 2.0 software (Psychology Software Tools, PA, USA). The participants viewed the pictures individually on a 13-inch laptop that was located approximately 60 cm from the subject. Initially, a video with instructions was presented to evaluate the emotional response to each picture on the SAM and PAD scales. The participants practiced the assessment procedure using five control pictures from the IAPS.

All of the pictures were presented only once in four counterbalanced blocks with 64 trials each, with the constraint of not presenting the same picture category consecutively. Each trial consisted of three parts: 6 seconds of picture presentation, picture rating using the SAM and PAD scales, and a 5-second intertrial interval. Each session lasted an average of 40 minutes.

### Statistical Analysis

Initially, the distribution of the 64 pictures was analyzed in the emotional dimensions using a scatterplot with two axes (valence vs. arousal). Pearson’s linear correlation was used to analyze correlations between appetitive valence and arousal and between aversive valence and arousal. To evaluate emotional and craving responses, we performed separate mixed 3 × 2 × 4 analyses of variance (ANOVAs) for the valence, arousal, dominance, and craving scales, with Age (adolescents, young adults, and adults) and Sex (male and female) as the between-subjects factors and Picture (pleasant, neutral, and unpleasant IAPS pictures and GHW pictures) as the within-subjects factor.

Subsequently, the emotional and craving responses to the four categories of GHWs (i.e., cancer, cardiovascular disease, abortion, and childhood illness) were analyzed using 3 × 2 × 4 ANOVAs, with Age and Sex as between-subjects factors and Picture (cancer, cardiovascular disease, abortion, and childhood illness) as the within-subjects factor. Greenhouse-Geisser correction was used to correct any violation of sphericity for the repeated measures. *Post hoc* analyses of mean values were performed using paired multiple comparisons, adjusted with Bonferroni correction.

Finally, to study the influence of sex, age, and smoking status on the evaluations of the GHWs, four multiple regression analyses were performed using the mean valence, arousal, dominance, and craving scale scores as dependent variables. The level of significance was set at *p* < .05. All of the statistical analyses were performed using SPSS 20.0 software.

## Results

### Emotional Impact of GHW and IAPS Pictures

**Figure 1:**
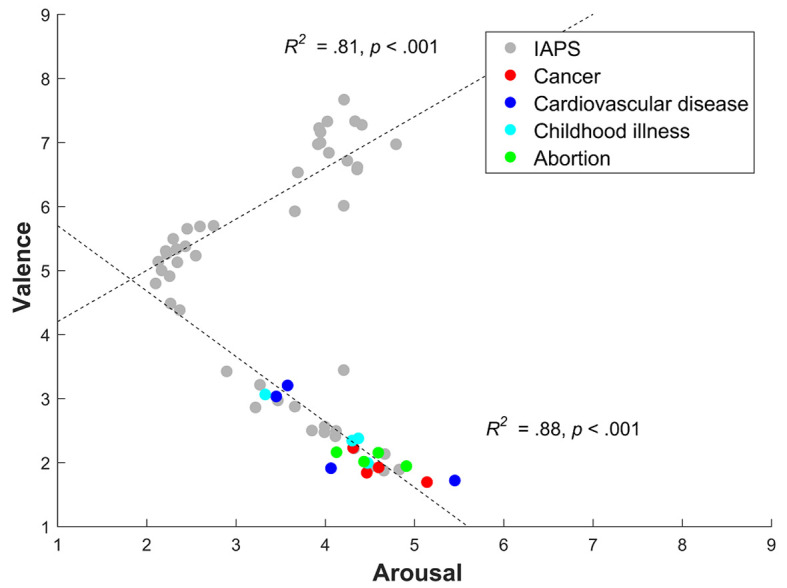
Distribution of Graphic Health Warnings (GHWs) and International Affective Picture System (IAPS) Pictures in the Two-Dimensional Affective Space (Valence and Arousal Dimensions)

Figure 1 shows the distribution of all of the pictures (i.e., IAPS and GHW) in the two-dimensional affective space, composed of valence (y-axis) and arousal (x-axis) ratings. As expected, the IAPS pictures are distributed in the form of a boomerang. Pictures that generated appetitive affective states (positive pole) and those that generated aversive affective states (negative pole) elicited moderate arousal, whereas neutral pictures generated low arousal. GHWs are located exclusively at the negative pole, indicating activation of the defensive motivational system (i.e., associated with avoidance behaviors), although with a moderate level of activation.

For all pictures, the quadratic correlation was significant (*R*^2^ = .80, *p* < .001). Pearson’s correlation was positive and significant between appetitive valence and arousal (*r* = .90, *p* < .001, *R*^2^ = .81) and negative and significant between aversive valence and arousal (*r* = −.94, *p* < .001, *R*^2^ = .88).

#### Valence Dimension

Table 2 shows the means and standard deviations for valence, arousal, dominance, and craving ratings. The ANOVA of the valence dimension revealed significant main effects of Picture, *F*(3, 882) = 1718.27, *p* < .001, $\eta_{p}^{2}$ = 0.85, and Sex, *F*(1, 294) = 4.44, *p* = .03, $\eta_{p}^{2}$ = 0.01. As expected, significant differences were found between pleasant, neutral, and unpleasant pictures (all *p* < .001). GHWs elicited more aversive affective states than unpleasant, neutral, and pleasant IAPS pictures (all *p* < .001). Women experienced more negative affective states than men (*p* = .03). However, as indicated by the significant Sex × Picture interaction, *F*(3, 882) = 5.66, *p* = .006, $\eta_{p}^{2}$ = 0.01, only unpleasant IAPS pictures and GHW pictures generated higher negative affective states in women than in men (both *p* < .02).

**Table 2:** Valence, Arousal, Dominance, and Craving Ratings

Emotional Dimensions and Craving	*M* (*SD*)					
	Women	Men	Adolescents	Young Adults	Adults	Total
Valence						
Pleasant	6.88 (1.05)	6.87 (1.10)	6.57 (1.11)	7.02 (0.91)	7.03 (1.12)	6.88 (1.07)
Neutral	5.26 (0.76)	5.10 (0.87)	5.13 (0.81)	5.13 (0.76)	5.25 (0.88)	5.17 (0.82)
Unpleasant	2.37 (0.82)	2.75 (1.03)	2.58 (0.97)	2.48 (0.97)	2.65 (0.93)	2.57 (0.96)
GHW	2.12 (1.01)	2.43 (1.21)	2.29 (1.15)	2.37 (1.09)	2.22 (1.16)	2.29 (1.13)
Arousal						
Pleasant	4.34 (1.99)	3.94 (1.79)	3.74 (1.90)	3.98 (1.98)	4.59 (1.71)	4.12 (1.89)
Neutral	2.54 (1.46)	2.17 (1.42)	2.30 (1.35)	2.28 (1.35)	2.42 (1.49)	2.34 (1.45)
Unpleasant	4.48 (1.82)	3.56 (1.68)	3.64 (1.88)	4.23 (1.71)	4.09 (1.78)	3.98 (1.80)
GHW	4.89 (2.10)	3.81 (1.99)	4.00 (2.08)	4.22 (1.94)	4.64 (2.24)	4.30 (2.11)
Dominance						
Pleasant	7.18 (1.62)	7.55 (1.43)	7.65 (1.49)	7.55 (1.46)	7.00 (1.56)	7.38 (1.53)
Neutral	7.76 (1.70)	8.20 (1.35)	8.07 (1.49)	7.90 (1.66)	8.02 (1.47)	8.00 (1.53)
Unpleasant	6.63 (1.78)	7.36 (1.54)	7.36 (1.77)	6.81 (1.58)	6.90 (1.68)	7.03 (1.69)
GHW	6.13 (1.97)	7.11 (1.67)	6.95 (1.81)	6.69 (1.87)	6.38 (1.92)	6.67 (1.88)
Craving						
Pleasant	1.51 (1.10)	1.64 (1.20)	1.29 (0.72)	1.47 (1.00)	1.93 (1.47)	1.58 (1.16)
Neutral	1.41 (0.94)	1.46 (0.97)	1.29 (0.84)	1.34 (0.86)	1.64 (1.09)	1.44 (0.96)
Unpleasant	1.49 (1.18)	1.65 (1.29)	1.28 (0.67)	1.41 (0.96)	1.99 (1.67)	1.58 (1.24)
GHW	1.40 (1.06)	1.49 (1.04)	1.28 (0.70)	1.32 (0.81)	1.72 (1.40)	1.45 (1.05)

*Note.* GHW = graphic health warning.

#### Arousal Dimension

For the arousal dimension, the ANOVA yielded significant main effects of Picture, *F*(3, 882) = 174.64, *p* < .001, $\eta_{p}^{2}$ = 0.37, and Sex, *F*(1, 294) = 14.85, *p* < .001, $\eta_{p}^{2}$ = 0.04, and a trend toward a significant main effect of Age, *F*(2, 294) = 2.82, *p* = .06, $\eta_{p}^{2}$ = 0.01. Pleasant and unpleasant IAPS pictures elicited higher levels of arousal compared with neutral IAPS pictures (both *p* < .001). GHWs generated higher levels of arousal than unpleasant and neutral IAPS pictures (both *p* < .001). Women experienced more arousal than men (*p* < .001). However, as indicated by the significant Sex × Picture interaction, *F*(3, 882) = 6.75, *p* = .001, $\eta_{p}^{2}$ = 0.02, only neutral and unpleasant IAPS pictures and GHW pictures generated higher arousal levels in women than in men (all *p* < .05). Adults experienced more arousal than adolescents (*p* = .05). However, as indicated by the significant Age × Picture interaction, *F*(6, 882) = 2.77, *p* = .01, $\eta_{p}^{2}$ = 0.01, only pleasant IAPS pictures generated higher arousal levels in adults than in adolescents (*p* = .006).

#### Dominance Dimension

The ANOVA of the dominance dimension revealed significant main effects of Picture, *F*(3, 882) = 95.71, *p* < .001, $\eta_{p}^{2}$ = 0.24, and Sex, *F*(1, 294) = 14.14, *p* < .001, $\eta_{p}^{2}$ = 0.04, a significant Age × Picture interaction, *F*(6, 882) = 3.27, *p* = .007, $\eta_{p}^{2}$ = 0.02, and a significant Sex × Picture interaction, *F*(3, 882) = 5.31, *p* = .003, $\eta_{p}^{2}$ = 0.01. Significant differences were found between pleasant, neutral, and unpleasant IAPS pictures (all *p* < .001). GHWs generated lower feelings of dominance compared with pleasant, neutral, and unpleasant IAPS pictures (all *p* < .001). Women experienced lower levels of dominance than men (*p* < .001). However, these differences were exclusive to neutral and unpleasant IAPS pictures and GHW pictures (all *p* < .02). Pleasant IAPS pictures elicited lower feelings of dominance in adults than in adolescents and young adults (both *p* < .04).

#### Craving Response

For craving, the ANOVA yielded significant main effects of Picture, *F*(3, 882) = 7.27, *p* < .001, $\eta_{p}^{2}$ = 0.02, and Age, *F*_2,294_ = 8.44, *p* < .001, $\eta_{p}^{2}$ = 0.05, and a significant Picture × Age interaction, *F*(6, 882) = 3.34, *p* = .004, $\eta_{p}^{2}$ = 0.02. All of the pictures produced low levels of craving, but GHWs and neutral IAPS pictures generated the lowest levels of craving compared with pleasant and unpleasant IAPS pictures (all *p* < .04). Adolescents experienced lower levels of craving than adults (*p* < .001). The Age × Picture interaction indicated that adolescents and young adults experienced lower levels of craving in response to pleasant and unpleasant IAPS pictures and GHW pictures than adults. Additionally, adolescents reported lower levels of craving in response to neutral IAPS pictures compared with adults (all *p* < .03).

### Emotional Impact of GHW Content

#### Valence Dimension

**Figure 2:**
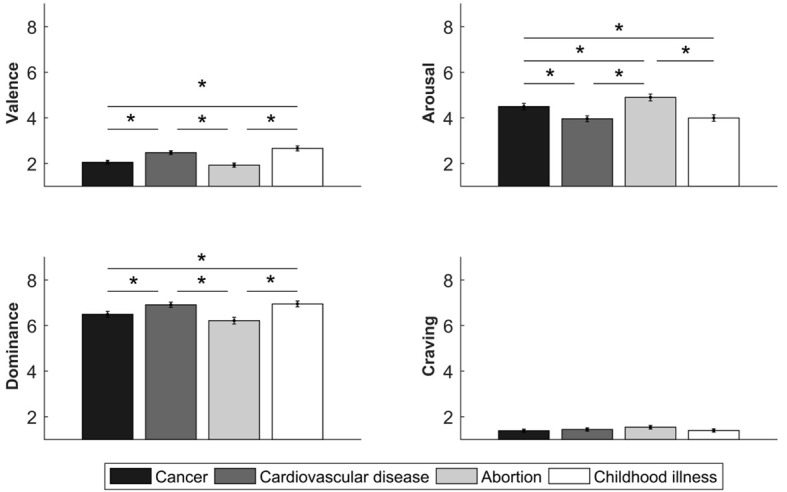
Valence, Arousal, Dominance and Craving Scores to Graphic Health Warning (GHW) *Note*. Bars indicate the standard error of the mean. *p < .05.

The ANOVA revealed significant main effects of Picture, *F*(3, 882) = 21.93, *p* < .001, $\eta_{p}^{2}$ = 0.06, and Sex, *F*(1, 294) = 6.15, *p* = .01, $\eta_{p}^{2}$ = 0.02. GHWs that depicted cancer and abortion generated higher unpleasant feelings than cardiovascular- and childhood illness-related GHWs (all *p* < .001; Figure 2). Generally, women (*M* = 2.11, *SE* = 0.09) experienced higher unpleasant feelings than men (*M* = 2.44, *SE* = 0.08, *p* = .01).

#### Arousal Dimension

The ANOVA yielded significant main effects of Picture, *F*(3, 882) = 26.26, *p* < .001, $\eta_{p}^{2}$ = 0.08, and Sex, *F*(1, 294) = 19.31, *p* < .001, $\eta_{p}^{2}$ = 0.06. GHWs that depicted abortion generated higher arousal compared with the other GHW categories (all *p* < .009). GHWs that depicted cancer elicited higher arousal than cardiovascular- and childhood illness-related GHWs (both *p* < .002; Figure 2). Women (*M* = 4.86, *SE* = 0.17) experienced higher feelings of arousal in response to GHWs compared with men (*M* = 3.81, *SE* = 0.16, *p* < .001).

#### Dominance Dimension

The ANOVA revealed significant main effects of Picture, *F*(3, 882) = 15.29, *p* < .001, $\eta_{p}^{2}$ = 0.04, and Sex, *F*(1, 294) = 19.96, *p* < .001, $\eta_{p}^{2}$ = 0.06. GHWs that depicted cancer and abortion generated lower feelings of dominance than cardiovascular- and childhood illness-related GHWs (all *p* < .005; Figure 2). Generally, women (*M* = 6.17, *SE* = 0.15) experienced lower feelings of dominance than men (*M* = 7.11, *SE* = 0.14, *p* < .001).

#### Craving Response

The ANOVA yielded a significant main effect of Age, *F*(2, 294 = 5.62, *p* = .004, $\eta_{p}^{2}$ = 0.03. GHWs produced low levels of craving in all of the participants, but adolescents (*M* = 1.27, *SE* = 0.10) reported lower levels of craving compared with adults (*M* = 1.71, *SE* = 0.10, *p* = .007).

### Influence of Sex, Age, and Smoking Status on GHW Ratings

For valence ratings, the stepwise regression yielded a significant model for Smoking status (β = 0.16, *R*^2^ = .02, *t* = 2.81, *p* = .005). Non-smokers experienced higher aversive feelings in response to GHWs. For arousal ratings, three significant models were found: Sex (β = −0.25, *R*^2^ = .06, *t* = −4.54, *p* < .001), Smoking status (β = −0.12, *R*^2^ = .07, *t* = −2.12, *p* = .03), and Age (β = 0.15, *R*^2^ = .10, *t* = 2.64, *p* = .009). Non-smoker adult women experienced higher feelings of arousal. For dominance ratings, a significant model for Sex was found (β = 0.26, *R*^2^ = .06, *t* = 4.65, *p* < .001). Women experienced less feelings of dominance than men. Finally, for craving ratings, two significant models were found: Smoking status (β = 0.38, *R*^2^ = .14, *t* = 7.20, *p* < .001) and Age (β = 0.13, *R*^2^ = .16, *t* = 2.41, *p* = .01). GHWs generated lower levels of craving in non-smoker adolescents.

## Discussion

The aim of the present study was to evaluate emotional (i.e., valence, arousal, and dominance) and craving responses to GHWs using stimuli that had a similar size as those that are used on actual cigarette packs. We also evaluated the influence of GHW content (i.e., images that depict cancer, abortion, cardiovascular disease, and childhood illness), sex, age, and smoking status on emotional responses and craving. The results showed that GHWs generated aversive affective states, with low arousal, high levels of dominance, and low craving. We also found that GHWs that depicted cancer and abortion had a greater emotional impact. Finally, our results suggested that GHWs have a greater emotional impact in non-smoker adolescent females.

Evaluation of the emotional impact showed that GHWs activated the defensive motivational system (i.e., aversive valence). Similar results were found with GHWs in Brazil (Nascimento et al., 2008) and the European Union (Muñoz et al., 2013). These results indicate that GHWs generally elicit affective states that are associated with avoidance behaviors (Bradley & Lang, 2007). GHWs are located on the cigarette package, thus favoring avoidance of the pack.

However, unlike the studies cited above, our results indicated that GHWs generated low levels of arousal and high levels of dominance, which can reduce their effectiveness because this decreases the probability of avoidance of the package. These results may be attributable to the fact that we used GHWs with a similar size as those on actual cigarette packages and also to a desensitization effect associated with the length of the exposure to these warnings, as previously described by Ratneswaran et al. (2014, 2016), and Woelbert and d’Hombres (2019). Regarding GHW size, previous studies showed that a smaller image size is associated with lower arousal that is generated by the picture (Codispoti & De Cesarei, 2007). Previous studies found that the minimum size of GHWs stipulated by WHO (i.e., 30% of the cigarette pack) is insufficient to have a significant emotional impact (Gantiva et al., 2016) and capture the attention of smokers and non-smokers (Gantiva et al., 2018). Altogether, these results suggest that increasing the size of GHWs can increase their emotional impact, especially the level of arousal, which also coincides with findings of previous studies (Scollo, Lindorff, Coomber, Bayly, & Wakefield, 2015; Wakefield et al., 2015; White, Williams, Faulkner, & Wakefield, 2015).

In the present study, the craving scores indicated that GHWs did not generate any desire to smoke, which is consistent with the objectives of WHO (2015) with regard to discouraging cigarette smoking. Low levels of craving and the aversive affective state that is generated by GHWs are necessary to compete with the positive affective state and high craving that are produced by stimuli that are associated with cigarettes (i.e., their packaging), especially in smokers (Cinciripini et al., 2006; Cui et al., 2012; Gantiva et al., 2015).

Our results also indicated that GHW content differentially impacts emotional responses. GHWs that depicted cancer and abortion generated more aversive valence, higher arousal levels, and lower dominance levels than cardiovascular- and childhood illness-related pictures. These results can be explained by activation of the defensive motivational system in response to pictures that represent explicit threat (Bradley, Codispoti, Cuthbert, & Lang, 2001) and suggest that GHWs that depict this kind of threat may more effectively generate an aversive emotional response compared with other GHW categories. Additionally, in Colombia abortion is not legalized, so messages related to this topic can generate a greater negative emotional response, which is reflected in a negative moral judgment (Haidt, 2001). Since only self-report measures were used in the study, cognitive assessment processes may also have been more clearly reflected.

Previous studies have shown that GHWs that show explicit damage have a greater emotional impact (Gantiva et al., 2016). In fact, in the study by Nascimento et al. (2008) that was conducted in Brazil, GHWs that depicted images of fetuses and newborn babies with diseases generated more aversive feelings compared with other picture categories. Warnings of cancer and abortion favor the use of images that are more explicit, and this content is more motivationally relevant because it represents a more direct threat, thus more reliably activating the defensive motivational system. Previous studies have also found that GHWs that negative valence and high arousal are associated with greater intention to quit smoking (Bekalu et al., 2019). It has also been shown that aversiveness could serve as one of the mechanisms through which GHWs produce desirable outcomes (Hammond, Fong, McDonald, Brown, & Cameron, 2004). Noar et al. (2015), developed a meta-analysis of GHWs experimental studies, they found that GHWs are more likely than text-only labels to produce reactance and aversiveness due possibly to the negative emotional reactions that such labels evoke.

Finally, the findings with regard to sex, smoking status, and age showed that sex was an important predictor of levels of valence, arousal, and dominance. In general, GHWs generated more negative affective states in women, with more arousal and lower feelings of dominance compared with men. These results are similar to Muñoz et al. (2013) and suggest that the design of GHWs for anti-tobacco campaigns should consider using warning images that target this group. Such measures may counteract the enormous efforts that tobacco companies have made in designing more appealing cigarette packages to increase women’s tobacco consumption (Carpenter, Wayne, & Connolly, 2005; Doxey & Hammond, 2011; Wakefield et al., 2002). Previous studies have also found that women rate GHWs as more effective than men (Tan, Bigman, Nagler, Minsky, & Viswanath, 2017). Other studies conducted only with women, have also found that participants reported looking more closely at the warnings on plain packs and thinking more about what the warnings were telling them (Moodie & Mackintosh, 2013).

The assessment of craving that was caused by GHWs showed that this feeling depends on both smoking status and age. Our results supported the hypothesis that GHWs can reduce the desire to smoke. Specifically, we observed lower levels of craving in adolescents and young adults compared with adults, regardless of the GHW content. Non-smoker adolescents exhibited the lowest desire to smoke among the studied groups. Accordingly, we consider that GHWs can be effective stimuli to discourage the initiation of smoking among youth, which is an expected outcome of the FCTC (WHO, 2005). A recent systematic review found that adolescents perceived GHWs as more effective than text-only warnings, with warnings depicting lung cancer, and oral diseases being perceived as particularly effective (Drovandi, Teague, Glass, & Malau-Aduli, 2019). GHWs have also been found to generate fear and guilt in adolescents, and these emotions increase the motivation to change in adolescent smokers (Netemeyer, Burton, Andrews, & Kees, 2016). GHWs that convey the risk of specific health effects from smoking can increase beliefs and knowledge about the health consequences of smoking, particularly for health effects that are lesser-known (Reid et al., 2017). Taken together, these results suggest that adolescents may benefit more from GHWs.

Interpretations of the present results should consider the following limitations. First, the measures of emotional impact involved only self-report instruments. Future investigations should use more objective measures of emotion (e.g., physiological responses, including facial electromyography, skin conductivity response, and electroencephalography). Second, the warnings that were assessed in the present study consisted only of images, and the emotional impact of text was not analyzed. Previous research showed that the use of text increases attention to the warning label (Brown, Reidy, Weighall, & Arden, 2013), but few studies have analyzed the emotional impact of such text. Third, the methodology that was employed in the present study should be replicated in other countries to confirm whether these results are generalizable to other cultures or societies. Nonetheless, the emotional response to stimuli that represent a threat has been reported to be quite stable across different countries. For example, United States (Lang et al., 2008), Spain (Vila et al., 2001), Mexico (Romo-González et al., 2018), Belgium (Verschuere et al., 2001), Colombia (Gantiva et al., 2011, Gantiva, Barrera-Valencia, et al., 2019), and Chile (Dufey et al., 2011). Finally, exposure to multiple stimuli containing GHW may lead to habituation effect which in turn might decrease the expected levels of valence and arousal. Future studies should consider reducing the number of GHW used as stimuli, and assess the corresponding emotional responses accordingly.

### Conclusions

Overall, the present study suggests that GHWs effectively reduce the attractiveness of cigarette packages, although their relatively small size (i.e., 30% of the cigarette package) decreases their effectiveness in generating avoidance behavior toward the package. The GHWs that were used in the present study did not generate craving, thus meeting the objective of decreasing the motivation to smoke cigarettes. With regard to GHW content, the present results suggest that images that depict cancer and abortion are the most effective in having an emotional impact that discourages smoking. Finally, the results suggest that GHWs have the greatest effect in non-smoker adolescent females.

## Footnotes

1 Pleasant pictures: 2071, 2075, 2151, 2155, 2347, 5829, 7405, 7461, 7492, 7660, 8163, 8186, 8191, 8208, 1731, 8158. Neutral pictures: 2235, 2393, 2493, 2595, 2745.1, 2980, 7018, 7026, 7036, 7038, 7041, 7161, 7179, 7493, 7021, 2579. Unpleasant pictures: 2688, 2981, 3195, 3213, 6415, 9185, 9187, 9321, 9341, 9435, 9904, 9908, 9922, 9940, 2095, 9332.

## Supplementary Materials

The Graphic Health Warnings (GHWs) used in the study are available via the PsychArchives repository (for access see Index of Supplementary Materials below).
